# Conditional ablation of vasopressin‐synthesizing neurons in transgenic rats

**DOI:** 10.1111/jne.13057

**Published:** 2021-11-08

**Authors:** Jun Watanabe, Yuki Takayanagi, Masahide Yoshida, Tatsuya Hattori, Michiko Saito, Kenji Kohno, Eiji Kobayashi, Tatsushi Onaka

**Affiliations:** ^1^ Department of Physiology Jichi Medical University Shimotsuke Tochigi Japan; ^2^ Institute for Research Initiatives Nara Institute of Science and Technology Ikoma Nara Japan; ^3^ Department of Organ Fabrication Keio University School of Medicine Tokyo Japan; ^4^ Present address: Bio‐science Research Center Kyoto Pharmaceutical University Kyoto Japan; ^5^ Present address: Department of Kidney Regenerative Medicine The Jikei University School of Medicine Tokyo Japan

**Keywords:** conditional ablation, diphtheria toxin, hypothalamic paraventricular nucleus, supraoptic nucleus, vasopressin

## Abstract

Vasopressin‐synthesizing neurons are located in several brain regions, including the hypothalamic paraventricular nucleus (PVN), supraoptic nucleus (SON) and suprachiasmatic nucleus (SCN). Vasopressin has been shown to have various functions in the brain, including social recognition memory, stress responses, emotional behaviors and circadian rhythms. The precise physiological functions of vasopressin‐synthesizing neurons in specific brain regions remain to be clarified. Conditional ablation of local vasopressin‐synthesizing neurons may be a useful tool for investigation of the functions of vasopressin neurons in the regions. In the present study, we characterized a transgenic rat line that expresses a mutated human diphtheria toxin receptor under control of the vasopressin gene promoter. Under a condition of salt loading, which activates the vasopressin gene in the hypothalamic PVN and SON, transgenic rats were i.c.v. injected with diphtheria toxin. Intracerebroventricular administration of diphtheria toxin after salt loading depleted vasopressin‐immunoreactive cells in the hypothalamic PVN and SON, but not in the SCN. The number of oxytocin‐immunoreactive cells in the hypothalamus was not significantly changed. The rats that received i.c.v. diphtheria toxin after salt loading showed polydipsia and polyuria, which were rescued by peripheral administration of 1‐deamino‐8‐d‐arginine vasopressin via an osmotic mini‐pump. Intrahypothalamic administration of diphtheria toxin in transgenic rats under a normal hydration condition reduced the number of vasopressin‐immunoreactive neurons, but not the number of oxytocin‐immunoreactive neurons. The transgenic rat model can be used for selective ablation of vasopressin‐synthesizing neurons and may be useful for clarifying roles of vasopressin neurons at least in the hypothalamic PVN and SON in the rat.

## Graphical abstract

We characterized a transgenic rat line that expresses a mutated human diphtheria toxin receptor under control of the vasopressin gene promoter. Intracerebroventricular administration of diphtheria toxin under a hyperosmotic condition induced selective ablation of vasopressin neurons in the hypothalamic paraventricular nucleus (PVN) and the supraoptic nucleus (SON) but not in the suprachiasmatic nucleus. Intrahypothalamic administration of diphtheria toxin induced selective ablation of vasopressin neurons in the injected areas of the PVN and SON.

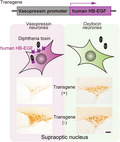

## 1 INTRODUCTION

Vasopressin, an evolutionally conserved peptide consisting of nine amino acids, shows similarities throughout the animal kingdom.^1^, ^2^, ^3^ Vasopressin not only has anti‐diuretic and vasopressor actions, but also affects social behaviors.^4^ The majority of vasopressin neurons are located within the hypothalamus including the hypothalamic paraventricular nucleus (PVN), supraoptic nucleus (SON) and suprachiasmatic nucleus (SCN). Vasopressin neurons in each brain region project to different target regions of the brain and are considered to exert different functions.^5^ To clarify region‐specific functions of vasopressin neurons, region‐specific experimental manipulation is considered to be effective. The human diphtheria toxin receptor shows more than 1000‐fold higher sensitivity to a diphtheria toxin than the receptor of rodents does.^6^ Selective induction of the human diphtheria toxin receptor in a certain type of cells of mice has been used for selective destruction of cells in a time‐dependent manner.^7^

Here, we report transgenic rats for which vasopressin neurons selectively express the human diphtheria toxin receptor. These rats were used to clarify the function of vasopressin neurons in the olfactory bulb.^8^ However, the detailed characteristics of these rats remain to be examined. We examined the expression of the diphtheria toxin receptor in the hypothalamus and investigated the effects of i.c.v. and intrahypothalamic injections of diphtheria toxin on the numbers of vasopressin neurons and oxytocin neurons. Lastly, the effects of destruction of hypothalamic PVN vasopressin neurons on social recognition, on object recognition memory and on social interaction were examined using the transgenic animal models because vasopressin receptors in the hippocampus have been shown to be involved in social recognition memory and, in addition, vasopressin neurons in the hypothalamic PVN project to the hippocampus.^9^

## 2 MATERIALS AND METHODS

### 2.1 Animals

Male Lewis rats (LEW/CrlCrlj) were obtained from Charles River Laboratories Japan, Inc. Transgenic rats expressing human heparin‐binding epidermal growth factor‐like growth factor (hHB‐EGF) as a diphtheria toxin receptor under control of the vasopressin promoter were created as described below. Male rats were used for all experiments. Rats were housed under a 12:12 h light/dark photocycle (lights on 7.30 pm) at 22 ± 2°C and 40%–70% relative humidity with food and water available ad lib. Rats were group‐housed before the surgical operation. After surgery, the rats were kept in individual cages. All animal procedures were approved by the Judging Committee of Experimental Animal Ethics of Jichi Medical University and were conducted in accordance with Japanese legislation concerning animal experiments.

### 2.2 Generation of transgenic rats

The plasmids p5‐VCAT‐3‐Sal and prVP8.2R1^10^ were kindly provided by Dr David Murphy (University of Bristol). The plasmid p5‐VCAT‐3‐Sal, a derivative of p5‐VCAT‐3,^11^ consists of the vasopressin structural gene containing a chloramphenicol acetyl transferase (CAT) cassette in exon III, flanked by 7 kbp of upstream and 3 kbp of downstream sequences. In p5‐VCAT‐3‐Sal, the translation start codon of the vasopressin gene was replaced with a unique *Sal*I site and another *Sal*I site at the 5′ end of the inserted gene was deleted and replaced with a *Not*I site. The plasmid prVP8.2R1 contains the entire vasopressin structural gene and 3 kbp of upstream and 3 kbp of downstream sequences. A CAT cassette in p5‐VCAT‐3‐Sal was replaced with a part of exon 2, whole intron 2 and exon 3, and 2.3 kbp of a downstream sequence in the vasopressin gene from prVP8.2R1 digested with *Sac*II. The plasmid pTRECK6^12^ includes hHB‐EGF complementary DNA (cDNA) and rabbit β‐globin and simian virus 40 polyadenylation signals. The 5′‐phosphorylated SalI linker sequence was ligated to the 5′ end of hHB‐EGF cDNA after partial digestion with EcoRI and fill‐in of 5′ end overhangs to form blunt ends with T4 DNA polymerase. To generate the transgene construct pVP‐hHB‐EGF, a 1.7‐kbp fragment digested with *Sal*I from pTRECK6, containing hHB‐EGF cDNA and β‐globin and simian virus 40 polyadenylation signals, was inserted into the original position of the modified translation start codon of the vasopressin gene in p5‐VCAT‐3‐Sal digested with *Sal*I and dephosphorylated.

**FIGURE 1:**
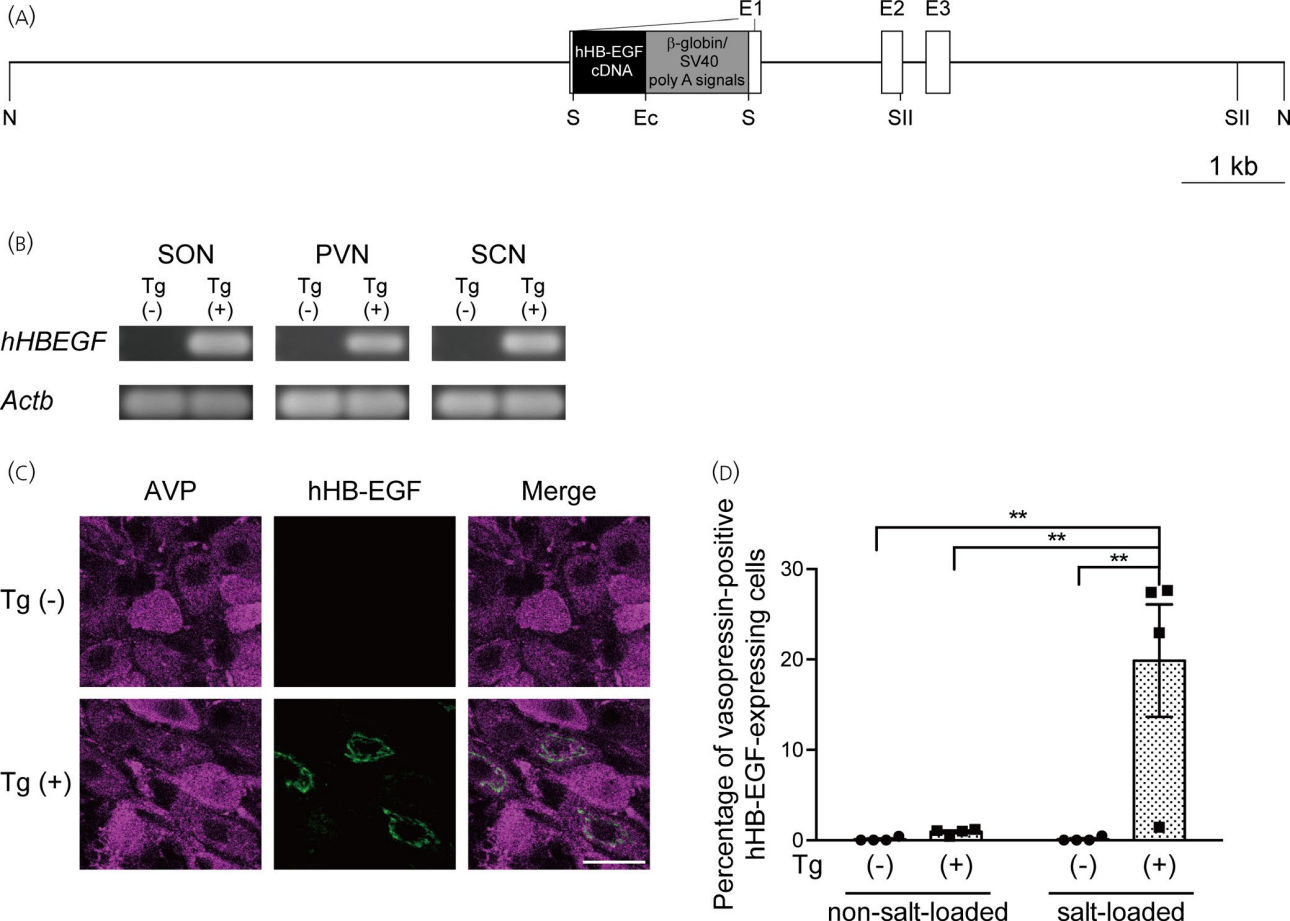
Generation of vasopressin‐human heparin‐binding epidermal growth factor‐like growth factor (hHB‐EGF) transgenic rats. (A) Structure of the transgene construct. The construct consists of the vasopressin structural gene, hHB‐EGF cDNA, rabbit β‐globin polyadenylation (poly A) signal and simian virus 40 (SV40) early gene polyadenylation signal. E1–E3, exon 1–exon 3. Restriction enzyme sites are shown below the transgene construct. Ec, *Eco*RI; N, *Not*I; S, *Sal*I; SII, *Sac*II. (B) Analysis of hHB‐EGF mRNA expression in the hypothalamic supraoptic nucleus (SON), hypothalamic paraventricular nucleus (PVN) and suprachiasmatic nucleus (SCN) of transgenic (Tg (+)) and wild‐type (Tg (‐)) rats by a reverse transcriptase‐polymerase chain reaction (RT‐PCR). The micropunched SON, PVN and SCN were evaluated for expression levels of hHB‐EGF mRNA by a RT‐PCR. Beta‐actin (*Actb*), a house‐keeping gene, was used as a positive control. (C) Immunostaining of the SON of salt‐loaded transgenic rats with anti‐vasopressin antibody (AVP; magenta) and anti‐hHB‐EGF antibody (hHB‐EGF; green). (D) Percentage of vasopressin‐immunoreactive cells expressing hHB‐EGF in the SON of salt‐loaded transgenic rats. **p < .01 vs. salt‐loaded wild‐type rats. Scale bar = 20 μm

The 12.3‐kbp *Not*I fragment from pVP‐hHB‐EGF (Figure 1A) was microinjected as a transgene into pronuclei of fertilized one‐cell embryos from Lewis rats (LEW/CrlCrlj; Charles River Laboratories Japan). These microinjected embryos were then transferred into pseudopregnant surrogate female rats. The transgenic founders were then mated with Lewis rats.

### 2.3 Reverse transcriptase‐polymerase chain reaction (RT‐PCR)

Brains were isolated immediately after decapitation of 7–8‐month‐old transgenic and wild‐type rats, frozen on dry ice and stored at −80°C. The hypothalamic part of the frozen brains was sectioned coronally at 300 μm in a cryostat at −10°C and then thaw‐mounted onto glass slides and refrozen. The regions containing the SON were micropunched from these sections that were kept frozen on dry ice using a 0.5‐mm‐diameter Sample Corer (Fine Science Tools) under a microscope. The regions containing the hypothalamic PVN and suprachiasmatic nuclei were micro‐punched from these sections kept frozen on dry ice using a 1.5‐mm diameter Harris Uni‐core (Ted Pella, Inc.) under a microscope. Punched tissues were stored in a nontoxic tissue RNA stabilization and storage reagent (RNAlater‐ICE; Thermo Fisher Scientific) at −20°C. RNA later‐stabilized tissues were taken from the reagent and were homogenized in a lysis buffer (RLT buffer; Qiagen GmbH) with an ultrasonic converter (Sonifier 250; Branson Ultrasonics Corporation). Total RNA was extracted from the homogenates with an RNeasy Protect Mini Kit (Qiagen) in accordance with the manufacturer's instructions.

Equal amounts (100 ng) of total RNA were transcribed into first strand cDNA for each sample using a SuperScript VILO cDNA Synthesis Kit (Life Technologies Japan Ltd) in accordance with the manufacturer's instructions and subjected to RT‐PCR.

The PCR conditions used for RT‐PCR were one cycle of 95°C for 5 min; 30 cycles of 94°C for 30 sec, 60°C for 30 sec and 72°C for 1 min; and one cycle of 72°C for 10 min. cDNA was used as template for PCR reactions using HotStarTaq Plus DNA Polymerase (Qiagen) to detect hHB‐EGF and β‐actin mRNAs as a positive control for the presence of cDNA in samples and for PCR amplification. Amplified RT‐PCR products were visualized by electrophoresis on 2% agarose gels stained with ethidium bromide.

The primers used were: 5′‐GGGACCCATGTCTTCGGAAATA‐3′ (forward) and 5′‐CCAGGATGGTTGTGTGGTCATAG‐3′ (reverse) for hHB‐EGF (GenBank accession no. NM_001945.3) and 5′‐GGAGATTACTGCCCTGGCTCCTA‐3′ (forward) and 5′‐GACTCATCGTACTCCTGCTTGCTG‐3′ (reverse) for β‐actin (GenBank accession no. NM_031144.3). All primer pairs were designed to span one intron. The expected sizes of PCR products for hHB‐EGF and β‐actin mRNA were 177 and 150 bp, respectively.

### 2.4 Droplet digital PCR

A QX200 droplet digital PCR system (Bio‐Rad Laboratories, Inc.) was used for determination of transgene copy number. Genomic DNA was digested with restriction enzymes, *Mse*I and *Sma*I. Rat oxytocin receptor gene was used as a reference gene for normalization of hHB‐EGF copy number. The assay was performed in a 20‐μL reaction volume containing ddPCR supermix (Bio‐Rad Laboratories, Inc.), hydrolysis probes and gene‐specific primers. The hydrolysis probes and PCR primers sets that were used were: rat oxytocin receptor forward: 5′‐AGGCCTACGTCACATGGATCAC‐3′, reverse: 5′‐AGCTGATGAGGCCGTAGCA‐3′ and TaqMan MGB probe: 5′‐VIC‐ACATTGTACCGGTCATC‐MGB‐3′ (Thermo Fisher Scientific) (GenBank accession no. NM_012871.3); hHB‐EGF forward: 5′‐CGGTGGTGCTGAAGCTCT‐3′, reverse: 5′‐CAGGCTCTCGCCAGTCAC‐3′ and Universal Probe Library probe #71 (Roche Applied Science).

### 2.5 Urine collection and measurement of urine osmolality and vasopressin concentrations

Rats i.c.v. injected with diphtheria toxin were housed in metabolic cages (3701M081; Tecniplast) for 6–8 days. After habituation in the cages for more than 1 day, urine was collected once per day and the volume was measured. Urine osmolality was measured by freezing point depression with an osmometer (Fiske Micro‐Osmometer Model 210; Fiske Associates) as described previously.^13^ Volume of water intake, volume of urine, urine osmolality and vasopressin concentrations were shown as an average of 3 consecutive days. Vasopressin concentrations were determined by radioimmunoassay with specific anti‐arginine vasopressin antibody, as described previously.^14^

### 2.6 Surgical operation for injections of diphtheria toxin

For i.c.v. injection of diphtheria toxin, a surgical operation of i.c.v. cannula implantation was performed in transgenic rats and wild‐type rats as described previously.^15^ Briefly, 13–27‐week‐old rats were anesthetized with Avertin (200 mg kg^–1^, i.p.; tribromoethanol; WAKO Pure Chemical Industries Ltd) and placed in a stereotaxic frame. A stainless steel guide cannula (23‐gauge) was inserted into the right lateral cerebral ventricle (tip coordinates: 0.6 mm caudal to the bregma, 1.5 mm lateral to the midline and 4.0 mm below the skull) and secured to the skull with screws and dental cement. The rats were allowed to recover for 1 week. In non‐salt‐loaded groups, transgenic rats and wild‐type rats were injected i.c.v. with diphtheria toxin (10 ng per 5 μL, once per day, 3 consecutive days; Sigma‐Aldrich Corp) via an inner cannula (30‐gauge). In experiments with salt loading, wild‐type rats and transgenic rats were salt‐loaded orally with 2% NaCl solution as drinking water for 8 days. Transgenic and wild‐type rats were injected i.c.v. with diphtheria toxin (10 ng per 5 μL, once per day) on 3 consecutive days of days 5–7 after initiation of salt loading. In experiments with a vasopressin V2 receptor agonist, transgenic rats were salt‐loaded for 6 days, injected i.c.v. with diphtheria toxin (10 ng per 5 μL, once per day) on 3 consecutive days of days 3–5 after initiation of salt loading and were implanted with osmotic pumps s.c. for administration of a vasopressin V2 receptor agonist as described below.

For intrahypothalamic injection of diphtheria toxin under a normal osmotic condition, transgenic rats and wild‐type rats at the age of 5 months were used. Transgenic rats and wild‐type rats were anesthetized with Avertin (200 mg kg^–1^, i.p.) and were placed in a stereotaxic frame. Diphtheria toxin (5 ng per 0.5 μL per site) was injected unilaterally at two positions (0.84 mm caudal to the bregma, 1.0 mm right to the midline, and 8.0 and 8.6 mm below the skull) via a plastic needle (MicroFil; 35 gauge; World Precision Instruments). Diphtheria toxin was diluted in artificial cerebrospinal fluid (138 mmol L^–1^ sodium chloride, 5 mmol L^–1^ potassium chloride, 1.5 mmol L^–1^ calcium chloride, 1 mmol L^–1^ magnesium chloride, 11 mmol L^–1^ sodium bicarbonate, 1 mmol L^–1^ sodium phosphate, pH 7.2). Two weeks after surgery, brains were obtained for immunocytochemical examination.

For local ablation of SON vasopressin neurons, diphtheria toxin was microinjected bilaterally into the SON in transgenic rats and wild‐type rats at the age of 19–21 weeks under a normal osmotic condition. Transgenic rats and wild‐type rats were anesthetized with Avertin (200 mg kg^–1^, i.p.) and were placed in a stereotaxic frame. Diphtheria toxin (0.04 ng per 0.1 μL per site for transgenic rats and 0.04 or 0.1 ng per 0.1 μL per site for wild‐type rats) was injected bilaterally into the SON (0.9 mm caudal to the bregma, 1.7 mm lateral to the midline and 9.4 mm below the skull) via a plastic needle (MicroFil; 35 gauge). Diphtheria toxin was diluted in artificial cerebrospinal fluid. Two weeks after surgery, brains were obtained for immunocytochemical examination.

For destruction of the PVN, diphtheria toxin (0.04 ng per 0.1 μL per site) or a vehicle (0.1 μL per site) was injected bilaterally into the PVN (1.7 mm caudal to the bregma, 0.4 mm lateral to the midline and 8.0 mm below the skull) of another series of transgenic rats at the age of 10–12 weeks. More than 2 weeks after surgery, behavioral experiments were performed and brains were obtained for immunocytochemical examination. Immunocytochemical experiments including cell counting and behavioral experiments including quantification were conducted by experimenters who were blind to treatments of the animals. The number of vasopressin neurons in two rats out of 13 rats of the group injected with diphtheria toxin was more than half that of control rats and so the behavioral data were analyzed excluding these two rats.

### 2.7 Implantation of an osmotic mini‐pump with 1‐desamino‐8‐d‐arginine‐vasopressin (DDAVP)

Transgenic rats were anesthetized with halothane (1.5–4%), 8 weeks after i.c.v. injection of diphtheria toxin under a salt loading condition. Osmotic pumps (MODEL2006; Alzet) were s.c. implanted and a vasopressin V2 receptor agonist (DDAVP; H‐7675; Bachem) dissolved in normal saline was continuously infused by the pumps (infusion rate: 1 ng 0.15 μL^–1^ h^–1^).

### 2.8 Immunohistochemical detection of vasopressin, hHB‐EGF or oxytocin

To determine whether hHB‐EGF was expressed in vasopressin neurons of transgenic rats, 5–7‐month‐old transgenic rats and wild‐type rats were salt‐loaded (2% NaCl in drinking water) for 7 days and were anesthetized with Avertin (200 mg kg^–1^, i.p., tribromoethanol) and perfused transcardially with heparinized saline (20 U mL^–1^), followed by 4% paraformaldehyde (PFA) in 0.1 M phosphate buffer (pH 7.4). The brains were removed, postfixed in 4% PFA overnight and transferred to 30% sucrose solution in 0.1 m phosphate buffer until tissues sank. The brains were frozen on dry ice and stored at –80°C. Coronal brain sections were cut at 30 μm with a freezing sledge microtome. Every fourth section of the hypothalamus was collected and processed for immunohistochemical detection of vasopressin and hHB‐EGF.

Sections were incubated with 10% normal donkey serum for 1 h at room temperature and then with a guinea pig anti‐(Arg^8^)‐vasopressin polyclonal antibody (dilution 1:160,000; T‐5048; Peninsula Laboratories, LLC.; RRID: AB_2313978) for 48 h at 4°C, followed by incubation with Alexa Fluor 568 goat anti‐guinea pig IgG (4 μg mL^–1^; A11075; Thermo Fisher Scientific Inc; RRID: AB_2534119) for 24 h at 4°C. Vasopressin‐immunolabeled sections were treated with 10% normal donkey serum and then incubated with a goat anti‐hHB‐EGF polyclonal antibody (0.2 μg mL^–1^; AF‐259‐NA; R&D Systems, Inc; RRID: AB_354429) for 48 h at 4°C, followed by incubation with Alexa Fluor 488 donkey anti‐goat IgG (dilution 1:400; 705‐545‐147; Jackson ImmunoResearch Laboratories, Inc; RRID: AB_2336933) for 24 h at 4°C. After incubation, sections were washed and mounted onto slides with Prolong Gold anti‐fade reagent (Invitrogen). The sections were observed using a confocal microscope (TCS SP5; Leica Microsystems).

For detection of vasopressin and oxytocin‐immunoreactive cells, brain sections were prepared as described above and processed as described previously.^16^ In brief, sections were incubated with a guinea pig polyclonal antibody against vasopressin (dilution 1:200,000; T‐5048) or a guinea pig polyclonal antibody against oxytocin (dilution 1:1,000,000; T‐5021; Peninsula Laboratories, LLC.; RRID:AB_518526) for 48 h at 4°C, followed by incubation with biotinylated goat anti‐guinea pig IgG (2 μg mL^–1^; BA‐7000; Vector Laboratories, Inc; RRID:AB_2336132) for 2 h and then with avidin‐biotinylated horseradish peroxidase complex (dilution 1:50; Vectastain Elite ABC HRP kit, PK6100; Vector Laboratories) for 30 min at room temperature. Immunoreactivity was visualized as a brown cytoplasmic precipitate using a 3,3′‐diaminobenzidine procedure. For estimation of the effects of intrahypothalamic injection of diphtheria toxin on destruction of vasopressin‐immunoreactive cells, sections containing the SCN (seven sections; from −0.60 mm to −1.32 mm posterior to the bregma), the parvocellular part of the PVN (12 sections; from −0.96 mm to −2.28 mm posterior to the bregma), the magnocellular part of the PVN (four or five sections; from −1.56 mm to −2.04 mm posterior to the bregma) and the SON (12 sections; from −0.36 mm to −1.68 mm posterior to the bregma) were examined on the diphtheria toxin‐injected side at intervals of 120 µm in each rat.

In other experiments, sections containing the SCN (eight sections; from −0.36 mm to −1.20 mm posterior to the bregma), PVN (12 sections; from −0.96 mm to −2.28 mm posterior to the bregma), SON (18 sections; from 0.00 mm to −2.04 mm posterior to the bregma) were examined at intervals of 120 µm in each rat. The sum of the numbers of vasopressin‐immunoreactive neurons or oxytocin‐immunoreactive neurons was counted in each brain region, the boundary of which was determined in accordance with a brain atlas.^17^

Omission of the vasopressin antibody resulted in the absence of staining. Pre‐absorption of the anti‐vasopressin antibody with excess vasopressin has been reported to abolish staining.^18^ Pre‐absorption of the anti‐oxytocin primary antibodies with an excess of oxytocin (100 μm) but not with an excess of vasopressin (100 μm) abolished the staining.^13^

### 2.9 Behavioral tests

#### 2.9.1 Social recognition test

A social recognition test was performed as described previously.^8^, ^13^ Briefly, a juvenile male Lewis rat (30–36 days old) was presented to an adult male as a stimulus for 4 min (training session) in the home cage (43 × 27 × 20 cm) of the test rat. After a 30 min‐interval, both the same stimulus rat (familiar) and another stimulus rat (novel) were presented for 4 min (test session). Social recognition was estimated using a “preference index” ([time investigating novel animal]/[time investigating familiar animal + time investigating novel animal] ×100).

#### 2.9.2 Object recognition test

Rats were exposed to a polyethylene terephthalate cuboid bottle (5.5 × 5.5 × 14 cm; 250 ml) or a laboratory glass bottle (diameter 6.5 cm, height 14 cm) for 4 min in their individual home cages (43 × 27 × 20 cm). After a 30‐min interval, a familiar bottle was presented with the other novel bottle (both glass and plastic bottles) for 4 min.^8^ The time spent for investigation of objects was measured during the 4‐min test and the preference index ([time investigating novel object]/[time investigating familiar object + time investigating novel object] × 100) was calculated.

#### 2.9.3 Home cage social interaction test

Social behavior between two rats in a familiar environment was tested with a system that automatically analyzes behavior in home cages. The system consisted of a cage (43 × 27 × 20 cm) with recycled paper bedding PC (CL‐4130; CLEA Japan, Inc.), a filtered cage top containing an infrared video camera and infrared light emitting diodes (O’Hara and Co., Ltd). Male Lewis rats from a supplier were kept in cages in groups. A diphtheria toxin‐injected transgenic or vehicle‐injected control transgenic rat (20–22 weeks old) was placed in the test cage in a pair with a native male Lewis rat (17–18 weeks old). Their behaviors were video‐monitored for 2 days. Images from each cage were captured at a rate of one frame per second. Social interaction was measured by counting the number of particles in each frame: two particles indicated that the rats were not in contact with each other and one particle indicated contact between the two rats.

### 2.10 Statistical analysis

All statistical analyses were performed using Prism (GraphPad Software Inc.). Behavioral parameters had the maximum cutting values of observation duration and were therefore analyzed using non‐parametric statistics. Other values were analyzed using parametric tests. The percentages of vasopressin‐immunoreactive neurons expressing hHB‐EGF in non‐salt‐loaded and salt‐loaded wild‐type and transgenic rats were analyzed using two‐way ANOVA with a post‐hoc Tukey's multiple comparison test. Amounts of water intake, urine volumes, urine osmolarity and urine vasopressin concentration were compared between diphtheria toxin‐injected transgenic rats and diphtheria toxin‐injected wild‐type rats using an *F* test followed by Student's or Welch's *t* test. In experiments with DDAVP in transgenic rats, data were analyzed using a paired *t* test between before and after treatment. Numbers of vasopressin‐immunoreactive or oxytocin‐immunoreactive cells in diphtheria toxin‐treated transgenic and control rats were analyzed using an *F* test followed by Student's *t* test to compare two groups or by one‐way ANOVA with a post‐hoc Tukey's multiple comparison test. Behavioral data in a social recognition test and object recognition test were analyzed using a Mann–Whitney *U* test. Percentages of contact behavior and locomotor activity in a home cage in the social interaction test were analyzed using two‐way repeated measures ANOVA. Error bars denote the SEM. *p* < .05 was considered statistically significant.

## 3 RESULTS

### 3.1 Generation of transgenic rats expressing hHB‐EGF under control of the vasopressin promoter

The sensitivity towards diphtheria toxin varies among mammalian species. Humans show high sensitivity, whereas rats have resistance to diphtheria toxin. For conditional ablation of vasopressin neurons, we created transgenic rats that expressed a mutated hHB‐EGF gene under control of the vasopressin promoter (Figure 1A). The mutated hHB‐EGF has a high affinity to diphtheria toxin, but little EGF‐like activity.^12^ Transgene copy number in these transgenic rats determined by droplet digital PCR was found to be 8–9 copies of transgene per haploid genome (8.61 ± 0.05, *n* = 13). The mutated hHB‐EGF mRNA was detected in the SON, hypothalamic PVN and suprachiasmatic nuclei of transgenic rats, in which vasopressin neurons were located (Figure 1B). Immunoreactivity for hHB‐EGF was also examined (Figure 1C). The transgene of the mutated hHB‐EGF was inserted into the downstream region of the vasopressin promoter, which is activated by salt‐loading. Immunoreactivity for hHB‐EGF was examined in rats under a normal osmotic condition and in rats that received 2% NaCl as drinking water for 1 week. In salt‐loaded transgenic rats, the percentage of vasopressin‐immunoreactive neurons expressing hHB‐EGF was significantly higher than the percentages in non‐transgenic wild‐type rats and non‐salt‐loaded transgenic rats (Figure 1D, non‐salt‐loaded wild‐type rats: 0.110 ± 0.110%; non‐salt‐loaded transgenic rats: 0.928 ± 0.166%; salt‐loaded wild‐type rats: 0.121 ± 0.121%; salt‐loaded transgenic rats: 19.856 ± 6.230%; *n* = 4 each) (significant effect of group *F*_1,12_ = 10.87, *p* = .0064; significant effect of salt loading *F*_1,12_ = 9.227, *p* = .0103; significant interaction *F*_1,12_ = 9.207, *p* = .0104; two‐way factorial ANOVA; non‐salt‐loaded wild‐type vs. salt‐loaded transgenic, *p* < .01; non‐salt‐loaded transgenic vs. salt‐loaded transgenic, *p* < .01; salt‐loaded wild‐type vs. salt‐loaded transgenic, *p* < .01, post‐hoc Tukey's multiple comparison test). These results suggest that the transgene is expressed in vasopressin neurons of transgenic rats.

### 3.2 I.c.v. injection of diphtheria toxin in transgenic rats

**FIGURE 2:**
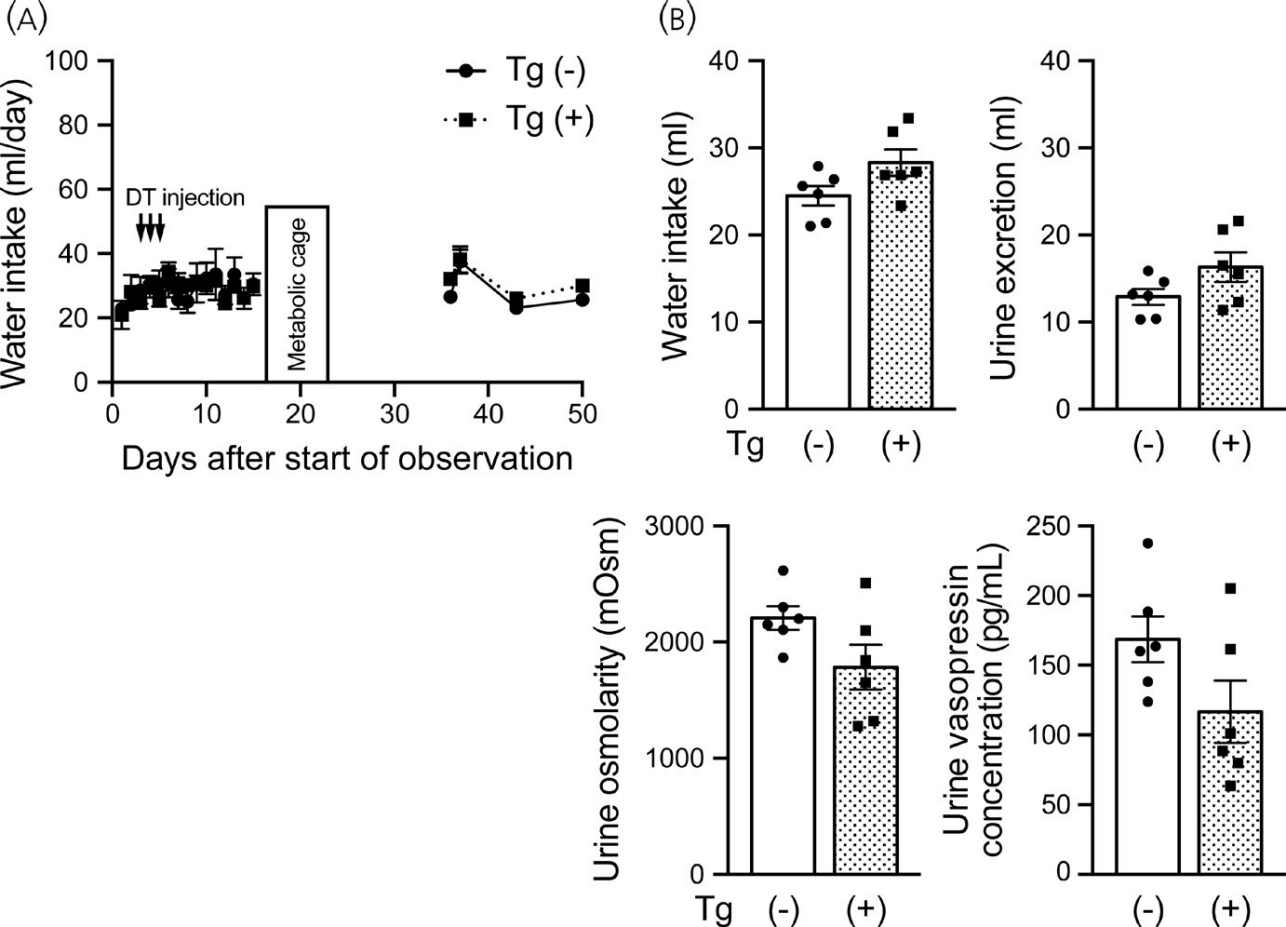
Effects of i.c.v. injection of diphtheria toxin on water intake, urine excretion, urine osmolarity and urine vasopressin concentration in transgenic rats. (A) Amounts of daily water intake in wild‐type and transgenic rats that received i.c.v. injections of diphtheria toxin (DT). (B) Amounts of water intake and urine excretion, urine osmolarity and urine vasopressin concentration in these rats during housing in metabolic cages. *n* = 6 each for wild‐type and transgenic rats. Data are presented as the mean ± SEM

We then investigated whether i.c.v. injection of diphtheria toxin ablated vasopressin neurons and influenced water homeostasis in transgenic rats. Diphtheria toxin was injected i.c.v. once a day for 3 days under a normal hydration condition and daily water intake was measured (Figure 2A). Amounts of water intake and urine excretion, urine osmolarity and urine vasopressin concentration were measured in metabolic cages 2 weeks after toxin injections (Figure 2B). No significant differences in water intake, urine excretion, urine osmolarity and urine vasopressin concentration were found between toxin‐injected transgenic rats and toxin‐injected wild‐type rats (water intake: *t*_10_ = 2.035, *p* = .0692; urine excretion: *t*_10_ = 1.764, *p* = .1083; urine osmolarity: *t*_10_ = 1.945, *p* = .0805, urine vasopressin concentration: *t*_10_ = 1.874, *p* = .0905, unpaired Student's *t* test).

### 3.3 I.c.v. injection of diphtheria toxin in salt‐loaded transgenic rats

**FIGURE 3:**
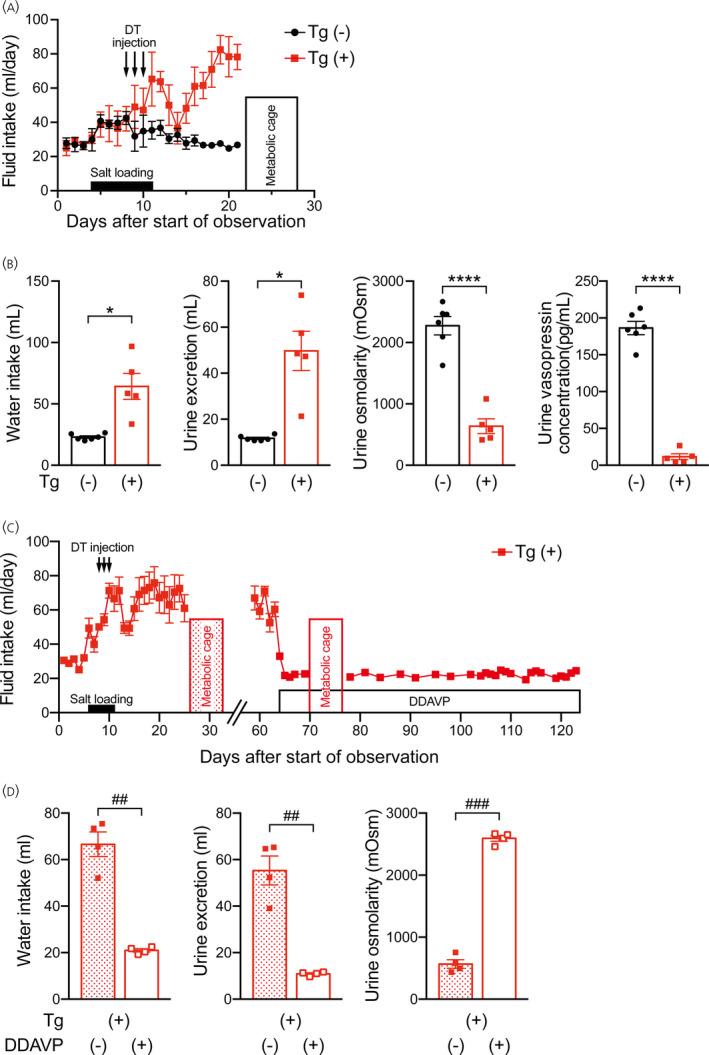
Effects of i.c.v. diphtheria toxin injection under the condition of salt loading on water intake, urine excretion, urine osmolarity and urine vasopressin concentration in transgenic rats. Diphtheria toxin (DT) was injected during the salt loading period. (A) Amounts of daily fluid intake in wild‐type (black) and transgenic (red) rats that received i.c.v. injections of diphtheria toxin under the condition of salt loading. (B) Amounts of water intake and urine excretion, urine osmolarity and urine vasopressin concentration in these rats during housing in metabolic cages. (C) Amounts of daily fluid intake in transgenic rats that received i.c.v. injections of diphtheria toxin under the condition of salt loading. The transgenic rats were treated with a vasopressin V2 receptor agonist (1‐desamino‐8‐d‐arginine‐vasopressin [DDAVP]) by an osmotic mini‐pump 8 weeks after injections of diphtheria toxin. (D) Amounts of water intake and urine excretion and urine osmolarity in these rats during housing in metabolic cages. *n* = 6 for wild‐type. *n* = 5 for transgenic rats (A, B) and *n* = 4 each for each group (C, D). *p < .05 and ****p < .0001 vs. wild‐type rats. ^##^ *p* < .01 and ^###^ *p* < .001, before vs. after treatment with DDAVP in transgenic rats. Data are presented as the mean ± SEM

To upregulate the expression of the transgene and to induce efficient ablation of vasopressin neurons, salt loading was applied in transgenic rats and in wild‐type rats before diphtheria toxin injection. Salt loading was applied by 2% NaCl drinking water. Diphtheria toxin was injected i.c.v. once a day for 3 days and daily water intake was measured (Figure 3A). Amounts of water intake and urine excretion, urine osmolarity and urine vasopressin concentrations were measured in metabolic cages 2 weeks after injection of the toxin. In transgenic rats i.c.v. injected with diphtheria toxin under a salt loading condition, water intake and urine excretion significantly increased and urine osmolarity and urine vasopressin concentration significantly decreased compared to those in wild‐type rats (water intake: *t*_4.078_ = 3.888, *p* = .0171, unpaired Welch's *t* test; urine excretion: *t*_4.024_ = 4.443, *p* = .0112, unpaired Welch's *t* test; urine osmolarity: *t*_9_ = 8.244, *p* < .0001, unpaired Student's *t* test; urine vasopressin concentration: *t*_9_ = 16.47, *p* < .0001, unpaired Student's *t* test) (Figure 3B). It is possible that deficiency in vasopressin synthesis and/or secretion as a result of toxin‐induced destruction of vasopressin neurons in these transgenic rats led to diabetes insipidus (polydipsia and polyuria). We then examined whether a vasopressin V2 receptor‐selective agonist (DDAVP) abolished these phenotypes. Diphtheria toxin was injected i.c.v. once a day for 3 days under the condition of salt loading, DDAVP was infused by an osmotic mini‐pump 8 weeks after injections of diphtheria toxin and daily water intake was measured in transgenic rats (Figure 3C). DDAVP infusion ameliorated polydipsia and polyuria in transgenic rats (water intake: *t*_3_ = 8.498, *p* = .0034; urine excretion: *t*_3_ = 7.077, *p* = .0058; urine osmolarity: *t*_3_ = 24.02, *p* = .0002, paired *t* test) (Figure 3D). In addition, the amount of water intake was increased again to 69.6 ± 18.7 mL day^–1^ in transgenic rats 65 days after implantation of DDAVP osmotic mini‐pumps as a result of depletion of DDAVP in the implanted pumps. From these results, phenotypes observed following i.c.v. injections with diphtheria toxin were considered to be caused by central diabetes insipidus.

### 3.4 Number of vasopressin neurons after i.c.v. injection of diphtheria toxin

To examine the specificity of destruction of vasopressin neurons in transgenic rats injected with diphtheria toxin, vasopressin immunoreactivity and oxytocin immunoreactivity in the brains of these rats were determined by immunohistochemistry.

The numbers of vasopressin‐immunoreactive neurons in the SON and PVN were greatly reduced in transgenic rats that received i.c.v. injections of diphtheria toxin under a salt loading condition compared to those in non‐salt‐loaded wild‐type rats or those in non‐salt‐loaded transgenic rats (SON: *F*_2,7_ = 121.2, *p* < .0001, one‐way ANOVA; *p* < .0001, salt‐loaded transgenic vs. non‐salt‐loaded wild‐type or non‐salt‐loaded transgenic rats, Tukey's multiple comparison test; PVN: *F*_2,7_ = 37.28, *p* = .0002, one‐way ANOVA; *p* < .001, salt‐loaded transgenic vs. non‐salt‐loaded wild‐type or non‐salt‐loaded transgenic rats, Tukey's multiple comparison test). The magnitudes of decrease compared to those in non‐salt‐loaded wild‐type rats were 97% for the SON and 88% for the PVN. The magnitudes of decrease compared to those in non‐salt‐loaded transgenic rats were 97% for the SON and 87% for the PVN.

On the other hand, the number of vasopressin‐immunoreactive neurons in the SCN was not significantly different among non‐salt‐loaded wild‐type rats, non‐salt‐loaded transgenic rats and salt‐loaded transgenic rats. (*F*_2,7_ = 0.7361, *p* = .5127, one‐way ANOVA).

**FIGURE 4:**
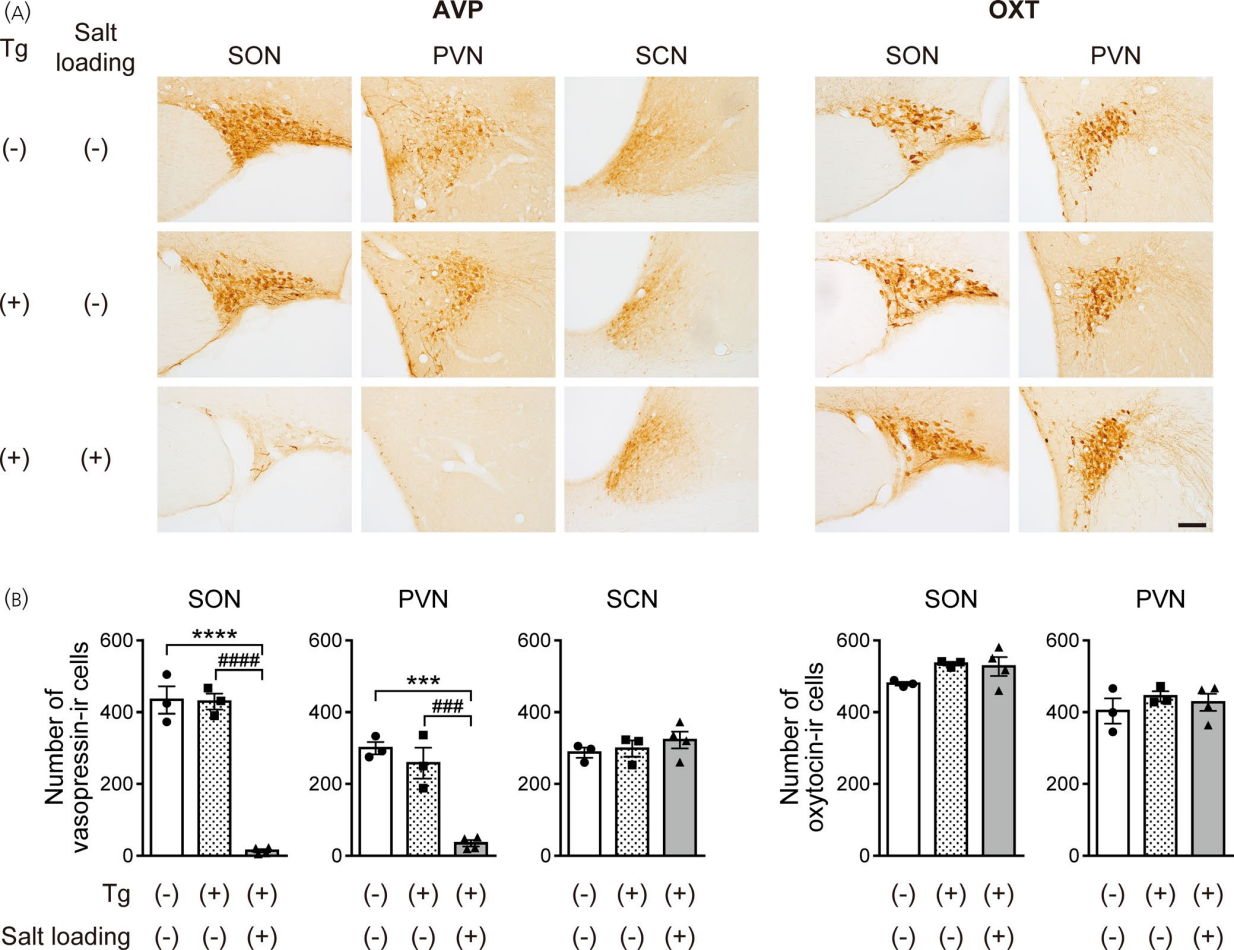
Ablation of vasopressin neurons in transgenic rats that received an i.c.v. injection of diphtheria toxin. (A) Photographs showing vasopressin (AVP) immunoreactivity (left) and oxytocin (OXT) immunoreactivity (right) in the supraoptic nucleus (SON), paraventricular nucleus (PVN) and suprachiasmatic nucleus (SCN) of transgenic rats i.c.v. injected with diphtheria toxin under non‐salt loading control or salt loading conditions. (B) Numbers of vasopressin‐immunoreactive (‐IR) or oxytocin‐IR cells in the SON, PVN and SCN of transgenic rats i.c.v. injected with diphtheria toxin. *n* = 3 each for non‐salt‐loaded wild‐type rats and non‐salt‐loaded transgenic rats and *n* = 4 for salt‐loaded transgenic rats. ***p < .001 and ****p < .0001, salt‐loaded transgenic rats vs. non‐salt‐loaded wild‐type rats. ^###^ *p* < .001 and ^####^ *p* < .0001, salt‐loaded transgenic rats vs. non‐salt‐loaded transgenic rats. Data are presented as the mean ± SEM. Scale bar = 100 μm

There were no differences in the numbers of oxytocin‐immunoreactive neurons in the SON and PVN among the three groups (number of oxytocin‐immunoreactive cells in the SON: *F*_2,7_ = 2.330, *p* = .1677, one‐way ANOVA; number of oxytocin‐immunoreactive cells in the PVN: *F*_2,7_ = 0.5690, *p* = .5902, one‐way ANOVA) (Figure 4). All of these findings suggest that upregulation of the transgene by salt loading is essential for effective ablation of vasopressin neurons in transgenic rats when diphtheria toxin is i.c.v. injected.

### 3.5 Number of vasopressin neurons following intrahypothalamic injection of diphtheria toxin

**FIGURE 5:**
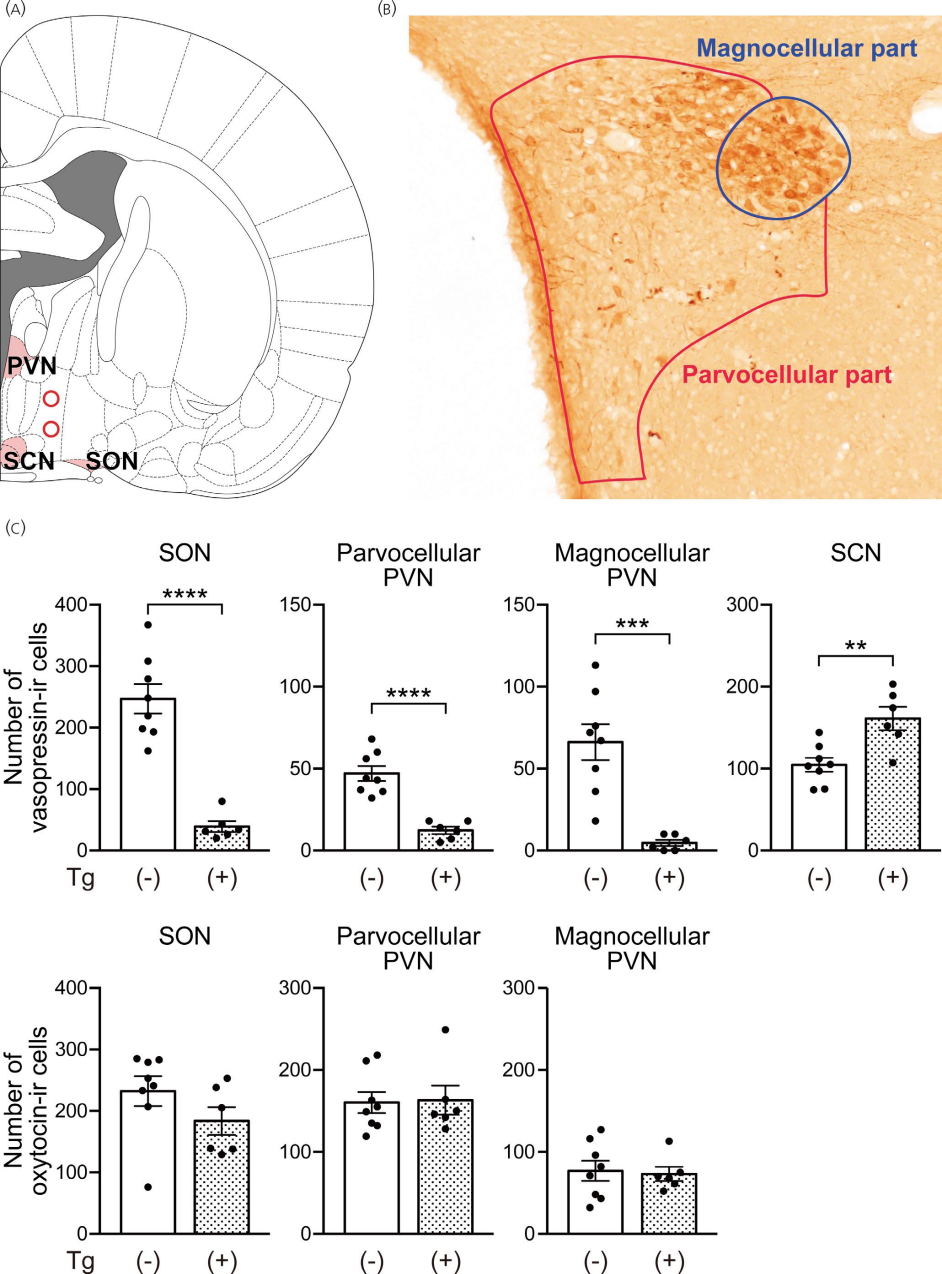
Ablation of vasopressin neurons by intrahypothalamic injection of diphtheria toxin in transgenic rats. (A) The injection sites (red circle) are indicated on the right hemisphere according to the coronal section from the Rat Brain Atlas.^17^ (B) The locations of parvocellular and magnocellular parts of the paraventricular nucleus (PVN) are drawn as lines on the photograph of vasopressin‐immunoreactive (‐IR) cells. (C) Numbers of vasopressin‐IR cells and oxytocin‐IR cells in the supraoptic nucleus (SON), the parvocellular and magnocellular parts of the PVN and the suprachiasmatic nucleus (SCN) of transgenic rats that received intrahypothalamic injection of diphtheria toxin. The numbers of vasopressin neurons on the toxin‐injected side were counted. *n* = 8 for control rats and *n* = 6 for transgenic rats. ***p < .001 and ****p < .0001 vs. wild‐type rats. Data are presented as the mean ± SEM

To examine whether injections of diphtheria toxin into brain parenchyma under a normal osmotic condition destroy vasopressin neurons in the PVN, SON and SCN at once, transgenic rats and wild‐type rats received unilateral intrahypothalamic injections of diphtheria toxin under a normal osmotic condition (Figure 5A). The numbers of vasopressin‐immunoreactive neurons in the SON, PVN and SCN on the diphtheria toxin‐injected side were counted. In these transgenic rats, the number of vasopressin‐immunoreactive neurons in the SON decreased by approximately 84% compared to that in wild‐type rats (*t*_8.770_ = 8.105, *p* < .0001; unpaired Welch's *t* test) (Figure 5C). The numbers of vasopressin‐immunoreactive neurons in the parvocellular part of the PVN of transgenic rats decreased by approximately 74% (*t*_12_ = 6.140, *p* < .0001; unpaired Student's *t* test) (Figure 5B,C) and those in the magnocellular part of the PVN of transgenic rats decreased by approximately 93% (*t*_7.419_ = 5.508, *p* = .0007; unpaired Welch's *t* test) (Figure 5B,C). The number of vasopressin‐immunoreactive neurons in the SCN of transgenic rats was significantly larger than that in wild‐type animals (*t*_12_ = 3.616, *p* = .0035; unpaired Student's *t* test) (Figure 5C). On the other hand, the numbers of oxytocin‐immunoreactive neurons in the SON and PVN were not significantly affected (number of oxytocin‐immunoreactive cells in the SON: *t*_12_ = 1.413, *p* = .1831; number of oxytocin‐immunoreactive cells in the parvocellular part of the PVN: *t*_12_ = 0.1367, *p* = .8935; number of oxytocin‐immunoreactive cells in the magnocellular part of the PVN: *t*_12_ =0.2301, *p* = .8219; unpaired Student's *t* test) (Figure 5B,C). These results showed that diphtheria toxin injection into brain parenchyma could destroy most of the parvocellular vasopressin neurons in the PVN as well as magnocellular vasopressin neurons in the SON and PVN in these transgenic rats under a normal osmotic condition.

### 3.6 Ablation of vasopressin neurons following microinjection of diphtheria toxin into the SON

**FIGURE 6:**
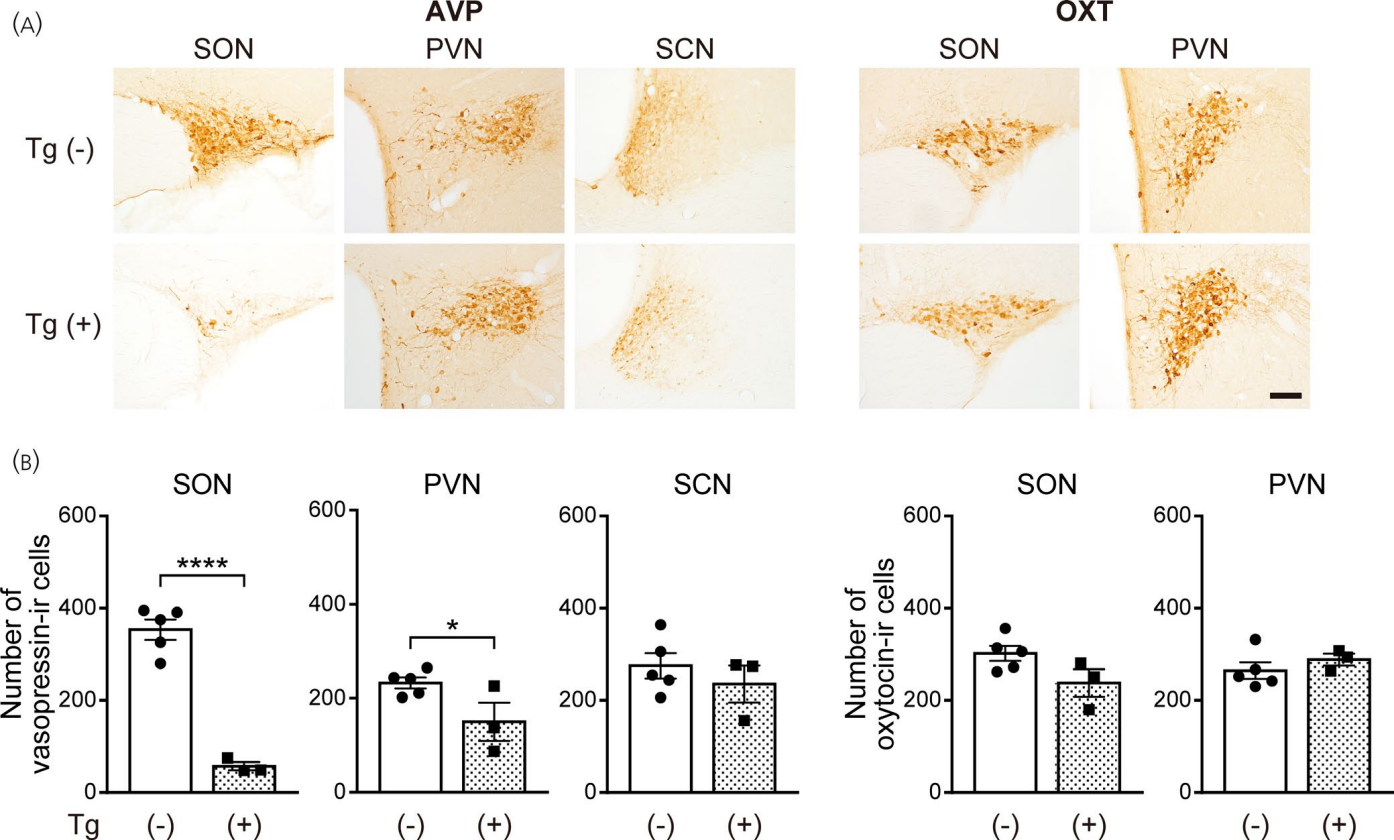
Specific ablation of vasopressin neurons in the supraoptic nucleus (SON) of transgenic rats locally injected into the SON with diphtheria toxin. (A) Photographs showing vasopressin (AVP) immunoreactivity (left) and oxytocin (OXT) immunoreactivity (right) in the SON, paraventricular nucleus (PVN) and suprachiasmatic nucleus (SCN) of wild‐type and transgenic rats injected into the SON with diphtheria toxin. (B) Numbers of vasopressin‐immunoreactive (‐IR) cells and oxytocin‐IR cells in the SON, PVN and SCN of transgenic rats injected into the SON with diphtheria toxin. *n* = 5 for control rats and *n* = 3 for transgenic rats. *p < .05 and ****p < .0001 vs. wild‐type rats. Data are presented as the mean ± SEM. Scale bar = 100 μm

For conditional ablation of vasopressin neurons in the SON, diphtheria toxin was microinjected into the SON of transgenic rats or wild‐type rats under a normal osmotic condition. In these transgenic rats, the number of vasopressin‐immunoreactive neurons in the SON decreased by approximately 84% compared to that in wild‐type rats (*t*_6_ = 9.824, *p* < .0001; unpaired Student's *t* test) (Figure 6). The number of vasopressin‐immunoreactive neurons in the PVN of transgenic rats also decreased by approximately 35% (*t*_6_ = 2.468, *p* = .0486; unpaired Student's *t* test) (Figure 6). On the other hand, the number of vasopressin‐immunoreactive neurons in the SCN and that of oxytocin‐immunoreactive neurons were not significantly affected (number of vasopressin‐immunoreactive cells in the SCN: *t*_6_ = 0.8424, *p* = .4319; number of oxytocin‐immunoreactive cells in the SON: *t*_6_ = 2.072, *p* = .0836; number of oxytocin‐immunoreactive cells in the PVN: *t*_6_ = 0.9367, *p* = .3850; unpaired Student's *t* test) (Figure 6). From these results, conditional ablation of vasopressin neurons was accomplished by local injection of diphtheria toxin without salt loading in transgenic rats.

### 3.7 Social behaviors in transgenic rats with destruction of vasopressin neurons in the PVN

**FIGURE 7:**
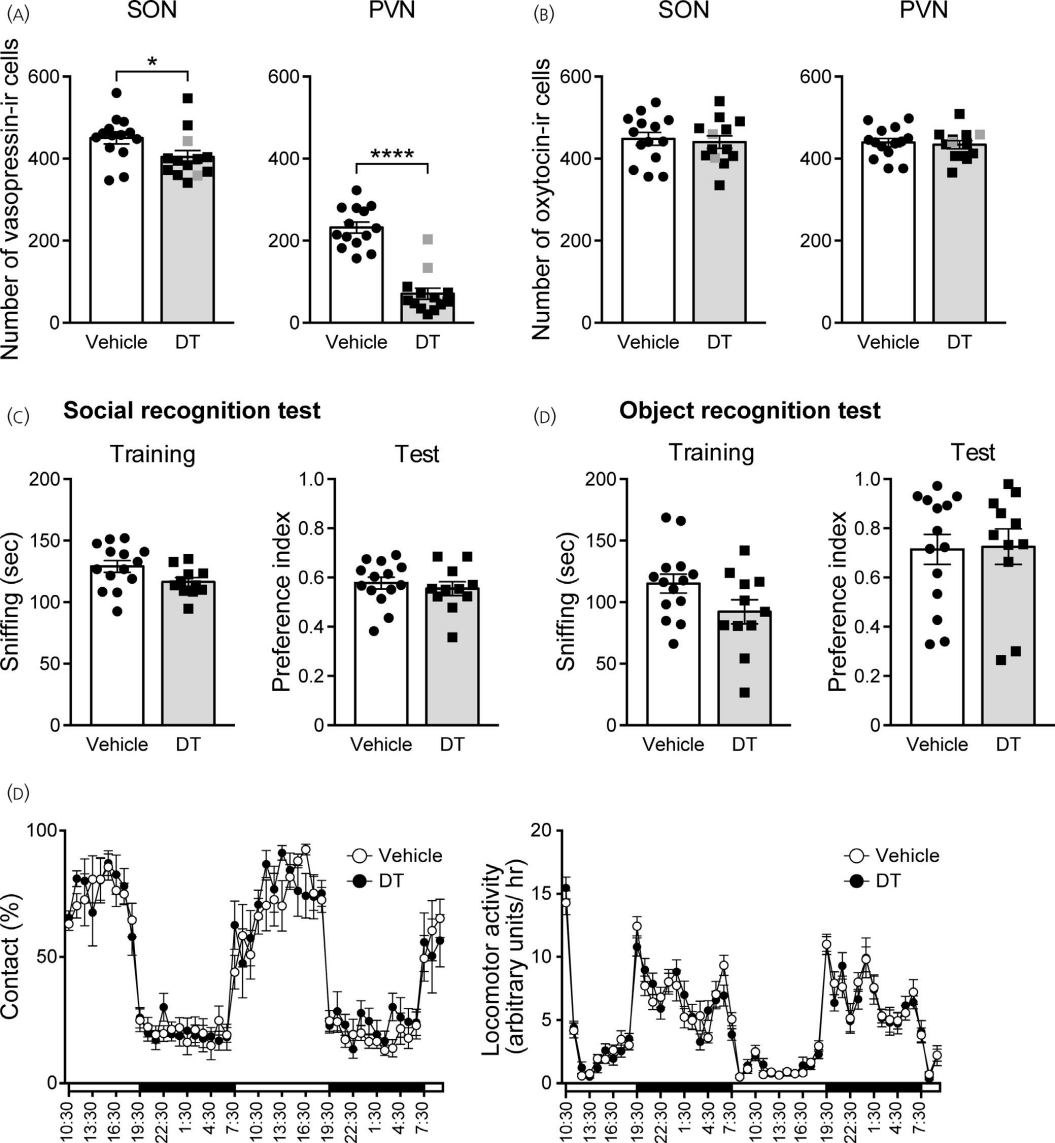
Social behavior test in transgenic rats locally injected into the paraventricular nucleus (PVN) with diphtheria toxin. (A) Numbers of vasopressin‐immunoreactive (‐IR) cells in the supraoptic nucleus (SON) and PVN of transgenic rats injected into the PVN with diphtheria toxin. The numbers of vasopressin neurons in two animals (as indicated by gray squares in Figure 7A) were more than half of the average of the number of vasopressin neurons in the control rats and these two rats were excluded from behavioral analysis to examine behavioral effects of ablation of PVN vasopressin neurons. (B) Numbers of oxytocin‐IR cells in the SON and PVN of transgenic rats injected into the PVN with diphtheria toxin. Gray squares indicate values of two rats excluded from behavioral analysis. (C) Social recognition test in transgenic rats that received ablation of vasopressin neurons in the PVN. (D) Object recognition test in transgenic rats that received ablation of vasopressin neurons in the PVN. (E) Home cage social interaction test in transgenic rats that received ablation of vasopressin neurons in the PVN. The percentage of time spent in contact (left) and locomotor activity (right) were evaluated. *n* = 14 for vehicle‐injected transgenic rats; *n* = 13 for diphtheria toxin‐injected transgenic rats (A, B); and *n* = 11 for vasopressin neuron‐ablated transgenic rats injected with diphtheria toxin (C–E). *p < .05, ****p < .0001 vs. vehicle‐injected transgenic rats. Data are presented as the mean ± SEM. DT, diphtheria toxin

Vasopressin in the hippocampus has been shown to play an important role in social memory.^9^ Infusion of antiserum for vasopressin into the hippocampus impairs social memory.^19^ The hippocampus receives vasopressin projections from the PVN, and opto‐genetical stimulation of vasopressin fibers from the PVN has been shown to facilitate social memory in a vasopressin receptor‐dependent manner.^20^ It is thus possible that PVN vasopressin neurons facilitate social recognition memory. Accordingly, we examined the effects of ablation of PVN vasopressin neurons on social memory. Diphtheria toxin was microinjected into the PVN of transgenic rats. The number of vasopressin‐immunoreactive neurons in the PVN was decreased by 70% and that in the SON was decreased by 10% compared to that in vehicle‐injected transgenic rats (number of vasopressin‐immunoreactive cells in the SON: *t*_25_ = 2.162, *p* = .0404; number of vasopressin‐immunoreactive cells in the PVN: *t*_25_ = 8.443, *p* < .0001; unpaired Student's *t* test) (Figure 7A). The numbers of oxytocin‐immunoreactive neurons in the SON and in the PVN were not significantly different between vehicle‐injected and diphtheria toxin‐injected transgenic rats (number of oxytocin‐immunoreactive cells in the SON: *t*_25_ = 0.3727, *p* = .7125; number of oxytocin‐immunoreactive cells in the PVN: *t*_25_ = 0.3802, *p* = .7070; unpaired Student's *t* test) (Figure 7B).

In two rats out of 13 rats of the diphtheria toxin‐injected group, the number of vasopressin neurons was more than half compared to that in vehicle‐injected rats. To determine the effects of ablation of PVN vasopressin neurons on behaviors, behavioral data were analyzed excluding these two rats. In a social recognition test, the time spent for sniffing a stimulus rat in the training session and preference index for a novel stimulus rat in the test session were not significantly different between vehicle‐injected rats and vasopressin neuron‐ablated rats (sniffing time: *U* = 44, *p* = .0726; preference index: *U* = 64, *p* = .5007; Mann‐Whitney *U* test) (Figure 7C).

We also examined object recognition memory as non‐social memory. In an object recognition test, the time spent for sniffing a stimulus object in the training session and preference index for a novel object in the test session were not significantly different between vehicle‐injected rats and vasopressin neuron‐ablated rats (sniffing time: *U* = 46, *p* = .0954; preference index: *U* = 75, *p* = .9358; Mann‐Whitney *U* test) (Figure 7D).

Vasopressin V1a receptor‐deficient mice have been reported to exhibit deficits in social interaction.^21^ Thus, we also examined social interaction in these rats. In a home cage social interaction test, we evaluated direct contact behavior between vehicle‐injected rats and intact Lewis rats and between diphtheria toxin‐injected rats and intact Lewis rats. The percentage of time spent in contact and locomotor activity were not significantly different between vehicle‐injected and diphtheria toxin‐injected rats (time spent in contact: time: *F*_47,1081_ = 35.06, *p* < .0001, group: *F*_1,23_ = 0.2815, *p* = .6008, interaction: *F*_47,1081_ = 0.8095, *p* = .8174; locomotor activity: time: *F*_47,1081_ = 48.44, *p* < .0001, group: *F*_1,23_ = 0.07499, *p* = .7867, interaction: *F*_47,1081_ = 0.9426, *p* = .5843; two‐way repeated measures ANOVA) (Figure 7E). Although vasopressin‐immunoreactive neurons in the PVN decreased by approximately 77% (Figure 6A), we did not find significant differences in social behaviors in these transgenic rats.

## 4 DISCUSSION

The human diphtheria toxin receptor, hHB‐EGF, has 1000‐fold higher sensitivity to diphtheria toxin compared to that of rodents, and selective induction of the receptor using a specific promoter has been employed for selective destruction of specific cells.^7^ Here, we characterized vasopressin‐human diphtheria toxin receptor transgenic rats. The human diphtheria toxin receptor was selectively expressed in vasopressin neurons of the transgenic rats. Intracerebroventricular injection of diphtheria toxin after salt loading selectively and almost completely destroyed vasopressin neurons in the PVN and SON and induced central diabetes insipidus (polydipsia and polyuria). Local injections of the toxin at a low dosage compared to i.c.v. injections (0.04 ng × 2 (both sides) vs. 10 ng × 3 times) decreased the number of vasopressin neurons selectively under a normal hydration condition compared to those in vehicle‐injected transgenic rats and toxin‐injected wild‐type rats. All of these findings suggest that the transgenic rat in the present study is a useful model for conditional destruction of vasopressin neurons in the PVN and SON and can be used for clarifying the role of vasopressin neurons in the PVN and SON.

In the present study, we could not detect significant expression of the human diphtheria toxin receptor in the SON or PVN of transgenic rats compared to wild‐type rats using an antibody under a normal hydration condition. On the other hand, mRNA for the human diphtheria toxin receptor was detected in the transgenic rats under a normal osmotic condition. Local injections of diphtheria toxin into the hypothalamus effectively destroyed vasopressin neurons in injection areas under a normal osmotic condition. Considering a previous study showing that a single molecule of diphtheria toxin that entered into the cell could effectively destroy the cell,^22^ it is likely that the sensitivity of immunocytochemical detection of the human diphtheria toxin receptor was not sufficiently high to detect the basal expression level of the receptor.

In the present study, i.c.v. injections of diphtheria toxin almost completely and selectively depleted vasopressin neurons in the PVN and SON after salt loading, indicating the effectiveness of the transgenic rat for selective destruction of vasopressin neurons in the PVN and SON. Although it remains to be clarified whether the human diphtheria toxin receptor is expressed in vasopressin neurons in other parts of the brain, direct injection into the olfactory bulb effectively blocked vasopressin functions in the olfactory bulb,^8^ suggesting the usefulness of the transgenic rat for destruction of vasopressin cells in other parts of the brain. However, in the present study, i.c.v. injections or intrahypothalamic injections of diphtheria toxin did not decrease the number of vasopressin neurons in the SCN, whereas diphtheria toxin receptor mRNA was found in the SCN. The amount of vasopressin mRNA has been shown to be smaller in the SCN than in the SON or PVN.^23^, ^24^ Thus, it is likely that the amount of the diphtheria toxin receptor expressed in the SCN was small compared with that in the SON or PVN and that the amount was not sufficient to induce destruction of vasopressin neurons in the SCN after diphtheria toxin injections under the present experimental conditions. The effectiveness of diphtheria toxin for destruction of vasopressin neurons in response to diphtheria toxin appeared to depend on brain regions and injection procedures. It is thus necessary to determine appropriate conditions of diphtheria toxin injections for effective destruction of vasopressin neurons of target brain regions in the present transgenic rat model.

In the present study, injection of diphtheria toxin into the PVN did not significantly affect social or object recognition memory, although the majority of vasopressin neurons in the PVN were ablated. It has been shown that vasopressin facilitates social recognition by acting on the lateral septum,^25^ as well as by acting on the hippocampus, and that the lateral septum receives vasopressin projections mainly from the medial amygdala and bed nucleus of the stria terminalis, suggesting the importance of vasopressin neurons in the medial amygdala and/or bed nucleus of the stria terminalis in the control of social recognition memory.

To clarify functions of vasopressin neurons in specific brain regions, it is useful to manipulate activity of vasopressin neurons in the regions. Transgenic rats^26^ and virus vectors^27^, ^28^ have been used for experimental manipulation of vasopressin neurons. In the present study, a transgenic rat line that expresses a transgene containing the construct of a mutated human diphtheria toxin receptor under control of the vasopressin gene promoter was found to express the human diphtheria toxin receptor in hypothalamic vasopressin neurons and to show selective destruction of vasopressin neurons in the PVN and SON after local injections of diphtheria toxin. This transgenic rat line can be used for selective destruction of vasopressin neurons to investigate functions of vasopressin neurons at least in the SON or PVN.

## CONFLICT OF INTERESTS

The authors declare that they have no conflicts of interest.

## AUTHOR CONTRIBUTIONS

**Jun Watanabe:** Investigation; Methodology; Writing – review & editing. **Yuki Takayanagi:** Conceptualization; Funding acquisition; Investigation; Methodology; Resources; Supervision; Writing – original draft; Writing – review & editing. **Masahide Yoshida:** Conceptualization; Funding acquisition; Investigation; Methodology; Resources; Supervision; Writing – original draft; Writing – review & editing. **Tatsuya Hattori:** Investigation; Methodology; Writing – review & editing. **Michiko Saito:** Methodology; Resources. **Kenji Kohno:** Methodology; Resources; Writing – review & editing. **Eiji Kobayashi:** Methodology; Resources. **Tatsushi Onaka:** Funding acquisition; Investigation; Methodology; Project administration; Resources; Supervision; Writing – original draft; Writing – review & editing.

## Data Availability

The data that support the findings of this study are available from the corresponding author upon reasonable request.
